# Machine Learning and In Vitro Chemical Screening of Potential α-Amylase and α-Glucosidase Inhibitors from Thai Indigenous Plants

**DOI:** 10.3390/nu14020267

**Published:** 2022-01-09

**Authors:** Tarapong Srisongkram, Sasisom Waithong, Thaweesak Thitimetharoch, Natthida Weerapreeyakul

**Affiliations:** 1 Division of Pharmaceutical Chemistry, Faculty of Pharmaceutical Sciences, Khon Kaen University, Khon Kaen 40002, Thailand; tarasri@kku.ac.th; 2 Human High Performance and Health Promotion Research Institute, Khon Kaen University, Khon Kaen 40002, Thailand; 3 Program of Aesthetic Sciences and Health, Graduate School, Faculty of Pharmaceutical Sciences, Khon Kaen University, Khon Kaen 40002, Thailand; namwansasi@gmail.com; 4 Division of Pharmacognosy and Toxicology, Faculty of Pharmaceutical Sciences, Khon Kaen University, Khon Kaen 40002, Thailand; thathi@kku.ac.th

**Keywords:** Thai indigenous plants, α-amylase, α-glucosidase, classification analysis, reinforcement learning, machine learning, anti-hyperglycemic, in vitro screening, multivariate analysis

## Abstract

Diabetes mellitus is a major predisposing factor for cardiovascular disease and mortality. α-Amylase and α-glucosidase enzymes are the rate-limiting steps for carbohydrate digestion. The inhibition of these two enzymes is clinically used for the treatment of diabetes mellitus. Here, in vitro study and machine learning models were employed for the chemical screening of inhibiting the activity of 31 plant samples on α-amylase and α-glucosidase enzymes. The results showed that the ethanolic twig extract of *Pinus kesiya* had the highest inhibitory activity against the α-amylase enzyme. The respective ethanolic extract of *Croton oblongifolius* stem, *Parinari anamense* twig, and *Polyalthia evecta* leaf showed high inhibitory activity against the α-glucosidase enzyme. The classification analysis revealed that the α-glucosidase inhibitory activity of Thai indigenous plants was more predictive based on phytochemical constituents, compared with the α-amylase inhibitory activity (1.00 *versus* 0.97 accuracy score). The correlation loading plot revealed that flavonoids and alkaloids contributed to the α-amylase inhibitory activity, while flavonoids, tannins, and reducing sugars contributed to the α-glucosidase inhibitory activity. In conclusion, the ethanolic extracts of *P. kesiya, C. oblongifolius, P. anamense*, and *P. evecta* have the potential for further chemical characterization and the development of anti-diabetic recipes.

## 1. Introduction

Diabetes mellitus (DM) has become one of the prevalent issues with the rising obesity crisis, leading to cardiovascular complications and mortality [1]. DM can be classified into two major types: type I DM (insulin-dependent) due to immune-mediated β cells destructions; and type II DM (non-insulin-dependent) due to an insulin secretory defect and insulin resistance [2]. Type II DM is the most common form of DM, accounting for 90–95% percent of DM diagnoses [1,3], caused by the impairment of insulin secretion by pancreatic β cells and the incapacity of tissues to use insulin [4]. The inability to produce or use insulin in the cells results in a high sudden surge of glucose levels in the bloodstream, called postprandial hyperglycemia. Long-term postprandial hyperglycemia leads to oxidative stress, produced by reactive oxygen species (ROS) [5]. The ROS can destroy the microvascular tissues, and increase the risk of micro-and macro-vascular complications, resulting in hypertension, myocardial infarction, diabetes retinopathy, dyslipidemia, and diabetes nephropathy [6]. Reducing postprandial hyperglycemia could prevent ongoing diseases and minimize the risk of micro-and macro-vascular complications [7]. Many approaches, such as medications, nutrient strategy, and lifestyle changes have been encouraged for reducing postprandial hyperglycemia for preventing insulin resistance and reducing cardiovascular risks [8].

The medication strategy is the primary approach for reducing sudden surge postprandial hyperglycemia. The mechanisms of action of antidiabetic medications are composed of (1) increasing insulin secretagogues, (2) lowering hepatic glucose production, (3) peroxisome proliferator-activated receptor-γ (PPARγ) agonists, (4) reducing glucose reabsorption by inhibiting sodium-glucose transport protein 2 (SGLT2) and (5) slow the rate of glucose absorption by inhibiting α-glucosidase [9]. These mechanisms are pivotal to slow the postprandial hyperglycemia [9].

The nutrient strategy effectively improves postprandial glycemia by preventing a sudden glucose uptake (or hyperglycemic peak) from the digestible polysaccharides [10]. α-Amylase and α-glucosidase are two enzymes that play a vital role in polysaccharides digestion. Inhibition of these two enzymes prevents the absorption of monosaccharides (e.g., glucose or maltose) and sudden high glycemic peaks that reduce insulin sensitivity [11]. Lowering glucose uptake by controlling the diet could benefit obese or overweight patients [10]. The acarbose, miglitol, and voglibose are synthetic compounds used in the clinic. These compounds, however, have certain adverse effects resulting in gastrointestinal (GI) disturbances (i.e., bloating, abdominal pain, GI cramping, or diarrhea) [10,12]. Natural products that could inhibit α-amylase and α-glucosidase activity are more promising than synthetic compounds (i.e., acarbose or voglibose) for lowering postprandial glycemia with fewer side effects such as bloating, abdominal pain, and diarrhea [10,11,13,14,15].

Natural products are a potential source of new drug design and drug discovery. The plants or natural products contain several active compounds against postprandial hyperglycemia [16]. Plant polyphenols (e.g., flavonoids, xanthones, tannic acids) can exhibit the anti-α-amylase and anti-α-glucosidase enzymes, which result in a reduction of the postprandial hyperglycemia [10,11,17,18,19,20]. Traditional Thai medicines (TTM) have long been used over decades for the treatment of diabetes mellitus [21]. Previous research found that TTM recipes in Northeastern and Southern Thailand, rich in polyphenols and tannins, could reduce postprandial hyperglycemia by inhibiting α-glucosidase and α-amylase enzymes [13]. The TTM in North and Northeastern Thailand, rich in saponin and reducing sugar, reduce postprandial hyperglycemia by inhibiting α-glucosidase activity [21]. The previous research points out that numerous species have long been used in Northeastern Thailand, but their underlying anti-diabetic activities, as well as their phytochemical constituents, have not yet been identified [13,22]. These findings may help adjust doses, and eventually result in personalized medicine. Therefore, identifying the anti-diabetic activities and phytochemical constituents of plants used in TTM are necessary to support these uses. The obtained information can be used as guidance for further phytochemical elucidation and drug discovery.

In this study, twenty-nine TTM plant species were selected based on the uses of Northeastern TTM practitioners in Thailand. Thirty-one samples from various parts of TTM plant species (i.e., *Abrus pulchellus* ssp. *mollis*, *Aganosma marginata*, *Artabotrys harmandii*, *Bombax anceps*, *Bridelia ovata*, *Cassytha filiformis*, *Catunaregam tomentosa*, *Cratoxylum formosum* spp. *pruniflorum*, *Croton oblongifolius*, *Diospyros castanea* (leaf)*, Diospyros castanea* (twig), *Diospyros winitii*, *Ellipeiopsis cherrevensis*, *Erythrophleum succirubrum*, *Erythroxylum cuneatum*, *Flacourtia indica*, *Garcinia cowa*, *Glochidion daltonii*, *Harrisonia perforate*, *Lannea coromandelica*, *Parinari anamense*, *Pinus kesiya*, *Polyalthia debilis*, *Polyalthia evecta* (leaf), *Polyalthia evecta* (rhizome), *Rhodamnia dumetorum*, *Rhus javanica*, *Rhus succedanea*, *Tetracera loureiri*, *Terminalia mucronata*, and *Terminalia triptera*) were collected and prepared as an ethanolic extract, and for the in vitro screening of α-glucosidase and α-amylase inhibitory activities. The presence of alkaloids, saponins, anthraquinones, reducing sugars, carotenoids, tannins, flavonoids, and xanthones was determined in the plant samples. The correlated-based network and multivariate analysis (i.e., principal component analysis (PCA) and supervised random forest classifier machine learning model) were used to study the phytochemical constituents associated with the α-amylase and α-glucosidase inhibitory activities. The results from this work enabled us to delineate which TTM plant samples had a high potential for being used for lowering postprandial glycemia, and which phytochemical components were responsible for α-amylase and α-glucosidase inhibitory activities.

## 2. Materials and Methods

### 2.1. Chemicals

The α-amylase (from porcine pancreas), α-glucosidase (from *Saccharomyces cerevisiae* Type I), dinitrosalicylic acid (98% purity), and 4–nitrophenyl α–D–glucopyranoside (99% purity) were purchased from Sigma-Aldrich (St. Louis, MO, USA). Monobasic sodium phosphate (99% purity), sodium chloride (99% purity), sodium hydroxide (97% purity), starch, and dibasic sodium phosphate (99% purity) were from Ajax Finechem Pty Ltd. (Auckland, New Zealand). Potassium sodium tartrate tetrahydrate (99% purity) was bought from Merck (Darmstadt, Germany). Dimethyl sulfoxide (99.7% purity) was purchased from Fisher Scientific (Loughborough, UK). Ethanol (99.9% purity) and methanol (99.9% purity) were bought from ACL Lab Scan Co. Ltd., (Bangkok, Thailand).

### 2.2. Plant Material and Extraction

Thirty-one plant samples from twenty-nine plant species were collected from four areas: two areas from Khon Kaen and another two areas from Chaiyaphum province, Northeastern Thailand. These were: (1) Khok Phu Ta Ka, Amphoe Phu Wiang; (2) Nam Phong district; (3) Chulabhorn Dam; and (4) Huai Kum Dam. The list of scientific names and plant parts used in this study are shown in Table 1. The plants were authenticated and taxonomically identified by their morphological features by Assistant Professor Thaweesak Thitimetharoch. The vouchers of the specimens were deposited at the herbarium of the Faculty of Pharmaceutical Sciences, Khon Kaen University, Thailand. The plants were cut into small pieces, air-dried, and then ground into coarse powder. The dried plants were extracted with 50% ethanol in water (1 g/6 mL) for seven days, with occasional shaking. The extract solvent was filtered and distilled using a rotary evaporator below 40 °C, and freeze-dried to yield the crude extract. The dry crude extracts were prevented from light, kept in an airtight container, and then in a desiccator for further use.

### 2.3. Phytochemical Identification

Qualitative phytochemical screening was performed in this study. The major phytoconstituents—flavonoids, tannins, anthraquinones, xanthones, carotenoids, saponins, alkaloids, and reducing sugar—in 0.05 g of plant extract were identified, as per the previous reports, with some modifications [23,24]. Briefly, for tannin and xanthones, plant extract (0.05 g) was dissolved in 2 mL of ethanol, incubated in a water bath (40 °C) for 10 min, centrifuged at 268× *g* for 10 min, and the supernatant collected.

For tannin, 2 mL of 15% FeCl_3_ was added, and a dark green or blue–black precipitate indicated the presence of tannin.

The xanthones were indicated by a yellow precipitate, after adding 100 µL of 5% potassium hydroxide to the supernatant.

For anthraquinones, the plant extract was dissolved in methanol, then the potassium hydroxide was added. The mixture was centrifuged at 268× *g* for 10 min. The pink color supernatant indicated the anthraquinone content.

For flavonoids, the sodium hydroxide was added into plant extract dissolved in 1 mL of methanol, then the 37% hydrochloric acid was added into the mixture of plant extract solution. The clear color change from the yellow color indicated the flavonoids content.

For alkaloids, the plant extract was dissolved in 1 mL of methanol and filtered. The supernatant was then added with 2 mL of 1% hydrochloric acid, followed by 1 drop of Dragendorff’s reagent (potassium bismuth iodide). A reddish-brown precipitate with turbidity indicated the presence of alkaloids.

For carotenoids, the plant extract was dissolved in 2 mL of chloroform and centrifuged at 268× *g* for 10 min. After that, the colorless supernatant was collected and added by 2 mL of concentrated sulfuric acid and heated for another 2 min. A blue or green blue color indicated the presence of carotenoids [25].

Saponins were indicated by permanent foam at 25 °C after the plant extract was dissolved in deionized water for 1 h, and shaken vigorously for 30 min [25].

Reducing sugar was indicated by the Fehling reagent mixture (Fehling A: Fehling B = 1:1). Fehling’s solution A was a mixture of 3.5 g of copper II sulfate in 50 mL deionized water; Fehling’s solution B comprised 5 g sodium hydroxide, 17.5 g potassium sodium tartrate, and 50 mL of deionized water. The plant extract was dissolved in 1 mL of de-ionized water, then heated with 1 mL of Fehling reagent mixture for 10 min. A brick-red precipitate indicated the presence of reducing sugar in the reducing sugar.

A positive result indicated that the existence of phytochemicals in the extracts were ranked from different levels of color intensity or precipitation formation and height of foam formation [26] compared with the control (without the crude extract). The positive result was ranked from high (5) to low (1), or absence (0). So, 0 was assigned when there was no change compared with the control. Moreover, the numbers 1, 2, 3, 4, and 5, were assigned for a slight change, a small change, a moderate change, more change, and the maximum change, respectively, compared with the control.

### 2.4. α-Amylase Inhibitory Activity

The plant extracts stock solution was prepared in DMSO and diluted with 0.06 M sodium phosphate buffer pH 6.9 to obtain various concentrations. The plant extract solution was added into the α-amylase solution in 0.06 M sodium phosphate buffer pH 6.9. This pH is the optimum pH for maximum enzyme activity. The solution mixture was incubated at 25 °C for 10 min, after that the 1% starch solution with dinitrosalicylic acid (DNS) solution was added and incubated at 100 °C for 10 min. The product of the enzymatic reaction was maltose, which reduced the pale, yellow-colored alkaline DNS to the orange–red-colored 3-amino-5 nitrosalicylic acid. This 3-amino-5-nitrosalicylic was measured at 540 nm using a UV-microplate reader (Sunrise, TECAN Austria GmbH). The percentage of enzyme inhibition was calculated by the percentage of the absorbance of sample minus blank, and divided by the control. The control was the reaction mixture containing starch, α-amylase, and DNS. Blank was the extract solution in the reaction mixture without the enzyme. The negative control was the reaction mixture containing DMSO and buffer solution instead of the extract solution. Under the concentration used there was no effect of DMSO on enzymatic reaction. The E_max_ was defined as the maximum efficacy (%inhibition) at the highest concentration of TTM (2.75 µg/mL). The IC_50_ was defined as the 50% inhibitory activity of the samples against the enzyme.

### 2.5. α-Glucosidase Inhibitory Activity

The plant extract solution (prepared as per Section 2.4) was added into the solution of α-glucosidase dissolved in the 0.1 M sodium phosphate buffer pH 6.9 and 0.1 M p-nitrophenyl-α-D-glucopyranoside, then incubated at 37 °C for 60 min. The products of the enzymatic reaction, α-D-glucopyranose and p-nitrophenol, were measured at 400 nm using a UV-microplate reader (Sunrise, TECAN Austria GmbH). The percentage of enzyme inhibition was calculated by the percentage of the absorbance of sample minus blank, and divided by the control. The control was the reaction mixture containing α-glucosidase, D-glucose, and p-nitrophenol. Blank was the extract solution in the reaction mixture without the enzyme. The negative control was the reaction mixture containing DMSO and buffer solution, instead of the extract solution. There was no effect of DMSO on the enzymatic reaction under the concentration studied. The E_max_ was defined as the maximum efficacy (%inhibition) at the highest concentration that the TTM extract could be dissolved in the solvent (0.67 µg/mL). The IC_50_ was defined as the 50% inhibitory activity of the samples against the enzyme.

### 2.6. Correlation-Based Network Analysis

The correlation-based network analysis between bioactivities (E_max_ and IC_50_ values against α-amylase and α-glucosidase) and the phytochemical constituents were performed by using Spearman’s correlation in NetworkX library [27] in Python version 3.9 software. The results from the Spearman’s correlation were represented as a correlation network, where each node represents each phytochemical constituent and the bioactivities based on the inhibition of α-amylase and α-glucosidase activities, and each edge represents the correlation score between two nodes. The respective green node and orange node represented each phytochemical constituent and each bioactivity. The node size represented the number of connections in that node. The respective positive and negative correlations (*r*) were presented in blue and red edges (the connection between two nodes). After the correlation-based network was constructed, the correlation-based network was further filtered with only significant correlation between each node by Spearman’s correlation analysis (*p* < 0.05).

### 2.7. Multivariate Classification Analysis

The multivariate classification was performed based on the IC_50_ bioactivity class including active (IC_50_ can be calculated) and inactive (IC_50_ cannot be calculated) classes against the α-amylase and α-glucosidase enzymes. The principal component analysis (PCA) was performed to normalize the phytochemical constituent ranking scores. The PCA standardization can improve the accuracy of the model when performing the multivariate analysis or machine learning (ML) task by: (1) eliminating correlated variables that do not contribute to any output of ML; (2) maximizing variances, which can improve the discrimination of the data, thus reducing the prediction error; and (3) simplifying the phytochemical components of original data into 2D, still containing all the phytochemical components from the original dataset. Then the PCA data and the accuracy score from the 5-fold cross-validation were used to select the classification model. The k-nearest neighbors (kNN), Naive Bayes (NB), support vector machine (SVM), random forest (RF), and decision tree (DT) were used for the model screening. The RF model was selected due to the higher classification performance (accuracy) of the training and the cross-validation (CV) model. The first RF model was built to select the best principal components (PC); then the top two PCs were chosen to build the second RF model. Finally, the decision boundaries were contrasted based on the classification of the top two PCs from the second RF model.

The performances of the model to classify the active and inactive between TTM samples were evaluated by the accuracy score (ACC), which can be calculated by Equation (1).

Accuracy = TP + TN/(TP + FN + TN + FP)(1)

The TP, TN, FP, and FN are true positive, true negative, false positive, and false negative, respectively. The area under the curves (AUC) of the receiver operating characteristic (ROC) of 5-fold cross-validation (CV) were also calculated to evaluate the sensitivity of the models. The sensitivity and specificity of the model were calculated by the true positive rate (TPR), and false positive rate (TPR), Equations (2) and (3).

TPR (sensitivity) = TP/(FP + FN)(2)

FPR (1 − specificity) = FP/(TN + FP)(3)

The machine learning model and multivariate analysis were built based on the software scikit-learn [28]. To promote reproducible research, all source codes about model building, network analysis, and statistical analysis can be found in https://github.com/TarapongSrisongkram/antihyperglycemia_Thaiherbs (accessed on 6 January 2022).

### 2.8. Statistical Analysis

Statistical analysis in this study was performed by using Python version 3.9 and SPSS software. Inhibition of enzymatic activities were experimentally performed in triplicate. All samples were tested for normality testing. The homogeneous subset analysis between IC_50_ and E_max_ values was performed by nonparametric Kruskal–Wallis with Mann–Whitney U post-hoc tests, where the *p*-values lower than 0.5 were considered as significantly different between-group. The correlation-based network analysis was analyzed by Spearman’s correlation, where the *p*-value lower than 0.05 was considered statistically significant.

## 3. Results

### 3.1. Screening of Phytochemical Constituents

**Figure 1:**
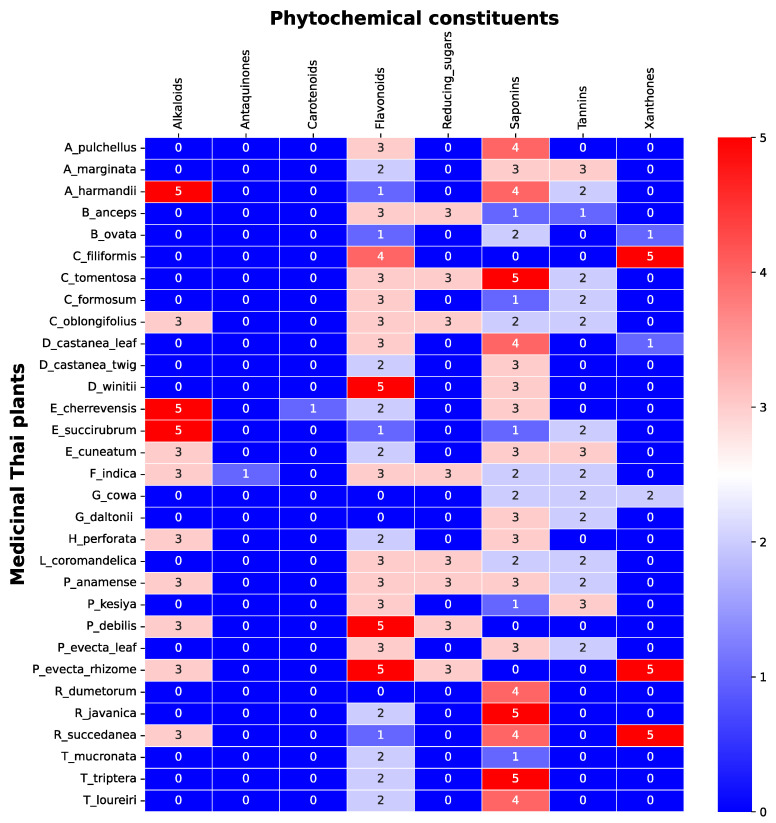
Phytochemical constituents of 31 TTM plant samples. The color bar of blue color to red color represents the amount, from not present (0 scores) to the highest presence (5 scores) of the phytochemical constituents in each sample.

The screening of phytochemical constituents is important to evaluate the attributed compounds of the bioactivity studied. The content of alkaloids, saponin, anthraquinones, reducing sugars, carotenoids, tannins, flavonoids, xanthones in 31 indigenous Thai plants were ranked as shown in Figure 1. Each plants comprised different types and amounts of phytochemicals. The amount of each phytochemical constituent was assigned between 0 to 5 (yellow to blue), where zero meant not found, 1 was the lowest, 2 was low, 3 was medium, 4 was high, and 5 was the highest amount of the phytochemical constituents found in the sample.

The highest amounts of alkaloids (5 scores) were found in *E. cherrevensis* and *A. harmandii*. The high amounts of alkaloids (4 scores) were found in *E. succirubrum*, then 3 scores were found in *P. debilis*, *P. evecta*, *E. cuneatum*, *A. marginata*, *F. indica*, *H. perforata*, *R. succedanea*, and *C. oblongifolius*.

The lowest amount of anthraquinones and carotenoids were found in *F. indica*, and *E. cherrevensis*, respectively. The highest amounts of flavonoids were found in *P. debilis*, *P. evecta*, and *D. winitii*. The high amounts of flavonoids were found in *C. filiformis*, and the medium amounts of flavonoids were found in *P. evecta* (leaf), *P. anamense*, *F. indica*, *P. kesiya*, *A. pulchellus*, *C. formosum*, *B. anceps*, *C. tomentosa*, *D. castanea* (leaf), *L. coromandelica*, and *C. oblongifolius*. The low amounts of flavonoids were found in *E. cuneatum*, *A. marginata*, *E. cherrevensis*, *H. perforata*, *T. loureiri*, *R. javanica*, *D. castanea* (twig)*, T. mucronata,* and *T. triptera*. The lowest amounts of flavonoid were found in *R. succedanea*, *E. succirubrum*, *B. ovata*, and *A. harmandii*.

The medium amount of reducing sugar was found in *P. debilis*, *P. evecta* (rhizome), *A. marginata*, *F. indica*, *B. anceps*, *C. tomentosa*, *L. coromandelica*, and *C. oblongifolius*. The highest amounts of saponins were found in *R. javanica*, *C. tomentosa*, and *T. triptera*. The high amounts of saponins were found in *R. dumetorum*, *A. pulchellus*, *T. loureiri*, *R. succedanea*, *D. castanea*, and *A. harmandii*. The medium amounts of saponin were found in *P. evecta* (leaf), *E. cuneatum*, *P. anamense*, *A. marginata*, *E. cherrevensis*, *H. perforata*, *G. daltonii*, *D. castanea*, and *D. winitii*. The low amounts of saponin were found in *G. cowa*, *F. indica*, *L. coromandelica*, *B. ovata*, and *C. oblongifolius*. The lowest amount of saponins were found in *P. kesiya*, *C. formusum*, *B. anceps*, *E. succirubrum*, and *T. mucronate*.

The medium amounts of tannins were found in the *E. cuneatum*, *A. marginata*, and *P. kesiya*. The low amounts of tannins were found in *G. cowa*, *P. evecta*, *A. marginata*, *C. formosum*, *G. daltonii*, *C. tomentosa*, *L. coromandelica*, *E. succirubrum*, *A. harmandii*, and *C. oblongifolius*. The lowest amounts of tannins were found in *B. anceps*.

The highest amounts of xanthones were found in the *P. evecta*, *C. filiformis*, and *R. succedanea*, and the lowest scores of xanthones were found in *D. castanea*, and *B. ovata*.

### 3.2. α-Amylase and α-Glucosidase Inhibitory Activities

The plant extracts were tested against α-glucosidase and α-amylase, and the results of 50% inhibitory concentration (IC_50_) and maximum effects (E_max_) were shown in Table 2. DMSO (%*v/v*) was used to dissolve the extract and it was maintained at a minimal state, so as not to denature the enzyme or have an interfering effect. The maximum concentrations of the TTM extract in α-amylase and α-glucosidase inhibitory activities were 2.75 µg/mL and 0.67 µg/mL, respectively. The E_max_ shows the maximum inhibition at the maximum concentration of the soluble plant samples. The plants that exerted a high E_max_ (≥90%) against α-amylase compared with other plants (*p* < 0.05) were *G. cowa* (99.6%), *P. debilis* (99.5%), *T. mucronata* (95.7%), *P kesiya* (94.9%), *P. evecta* (93.9%), *D. winitii* (93.3%), and *B ovata* (92.3%). The plants that exerted a high E_max_ (≥50%) against α-glucosidase compared with other samples (*p* < 0.05) were *P. anamense* (54.5%), *C. oblongifolius* (53.0%), *P. kesiya* (52.3%), *P. evecta* (leaf) (52.0%), *G. cowa* (51.1%), *P. evecta* (rhizome) (50.6%), *C. formosum* (50.3%), and *P. debilis* (50.0%). IC_50_ can be calculated for 13 plants indicating their activity from high to low against α-amylase, i.e., *P. kesiya*, *D. winitii*, *G. cowa*, *P. debilis*, *P. evecta* (leaf), *R. javanica*, *D. castanea* (leaf), *T. mucronata*, *B. ovata*, *F. indica*, *E. cuneatum*, *C. formosum*, and *E. cherrevensis*. Moreover, eight plants were active against α-glucosidase from high to low activity, i.e., *C. oblongifolius*, *P. anamense*, *P. evecta* (leaf), *P. kesiya*, *G. cowa*, *P. evecta* (rhizome), *C. formosum*, and *P. debilis*. We also found that five plants exhibited inhibition against both α-amylase and α-glucosidase, which were *P. evecta* (leaf), *P. kesiya*, *G. cowa, C. formosum*, and *P. debilis*. In addition, *P. kesiya* exerted the lowest IC_50_ values (0.11 µg/mL), indicating the highest activity against α-amylase compared with other plants (*p* < 0.05). On the other hand, *C. oblongifolius* (0.49 µg/mL), *P. anamense* (0.52 µg/mL), and *P. evecta (leaf)* (0.57 µg/mL) were the plant groups that exerted low IC_50_ values against α-glucosidase compared with other plants (*p* < 0.05) (Table 2).

### 3.3. Correlation-Based Network Analysis

**Figure 2:**
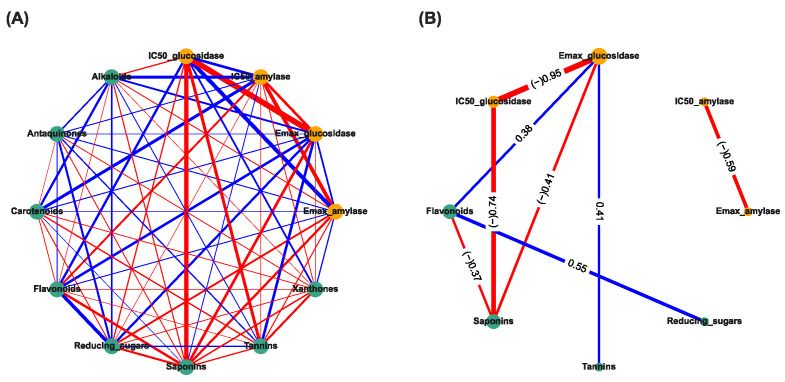
Correlation-based network analysis between α-amylase and α-glucosidase inhibitory activities and phytochemical components of 31 TTM plants samples. (**A**) Spearman’s correlation analysis between α-amylase and α-glucosidase inhibitory activities (i.e., E_max_ and IC_50_ against α-amylase and α-glucosidase enzymes) and eight phytochemical constituents found in 31 TTM samples. (**B**) Significance Spearman’s correlation between the α-amylase and α-glucosidase inhibitory activities and phytochemical compounds of 31 medicinal Thai herb samples (*p* < 0.05). The IC_50_ was categorized as active: 1 (IC_50_ can be calculated), and inactive: 0 (IC_50_ cannot be calculated). Each green node and orange node determines the phytocomponents, and bioactivities of the TTM, respectively. The respective positive (+) and negative (−) correlations (*r*) are presented in light-blue and red edges (connection line between two nodes). The thickness of each edge represents Spearman’s correlation coefficient (*r*).

The correlation-based network analysis between α-amylase inhibitory activities, α-glucosidase inhibitory activities, and phytochemical constituents are shown in Figure 2. The IC_50_ has been categorized into active: 1 (IC_50_ can be calculated), and inactive: 0 (IC_50_ cannot be calculated). The Spearman’s correlation coefficients (*r*) between anti-diabetic activities (yellow nodes) and phytochemical constituents (green nodes) were calculated and presented as the thickness of the edge between two nodes (Figure 2A). The significant correlation (*p* < 0.05) is shown in Figure 2B. The numeric results containing both correlation (r) and *p*-values can be found in the Supplementary Table S1. The results showed that the E_max_ of TTM against α-amylase was strongly negatively correlated with the active IC_50_ against α-amylase (*r* = −0.59, *p* < 0.05) (Figure 2B). The E_max_ against α-glucosidase were highly negatively correlated with the active IC_50_ against α-glucosidase (*r* = −0.95, *p* < 0.05), weakly negatively correlated with the quantities of saponins (*r* = –0.41, *p* < 0.05), and weakly positively correlated with tannins (*r* = +0.41, *p* < 0.05) and flavonoids (*r* = +0.38, *p* < 0.05), whereas the flavonoids were moderately positively correlated with the reducing sugar (*r* = +0.55, *p* < 0.05). In addition, the saponins were highly negatively correlated with the IC_50_ against α-glucosidase (−0.74, *p* < 0.05) and weakly negatively correlated with the quantities of flavonoids (*r* = −0.37, *p* < 0.05) (Figure 2B).

The results indicate that the E_max_ and IC_50_ against α-amylase enzymes were not significantly associated with the amounts of these eight phytochemical constituents, whereas the E_max_ against α-glucosidase was significantly directly associated with the quantities of flavonoids, tannins, and saponins, and indirectly weakly associated with the quantities of reducing sugars by flavonoids. The results suggest that the high amounts of flavonoids, tannins, and reducing sugars could increase the E_max_ (weak positive correlation) and yield the active IC_50_ (IC_50_ can be calculated) against the α-glucosidase enzyme, whereas a high amount of saponins could reduce the E_max_ (weak negative correlation) and yield the inactive IC_50_ against the α-glucosidase enzyme. This was confirmed as it was significantly directly negatively associated between the IC_50_ against the α-glucosidase and the quantities of saponins.

### 3.4. Classification Analysis

**Figure 3:**
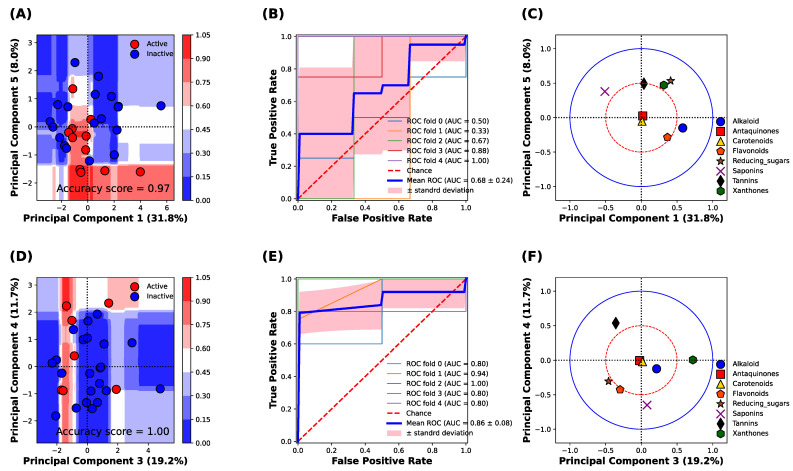
Classification analysis based on the random forest model of 31 TTM plant samples against α-amylase and α-glucosidase enzymes. (**A**) Decision boundary and score plot between principal component 1 (PC1) and component 5 (PC5) to predict the α-amylase inhibitory activity. Blue and red surfaces or dots indicate the inactive and active α-amylase inhibitory activity. (**B**) Receiving operating characteristic (ROC) of 5-fold cross-validation on the prediction of amylase inhibitory activity. (**C**) Loading plot of principal component analysis between PC1 to PC5. The blue solid circle indicates the correlation of the component in PC1 and PC5 at 1.0, and the red dash circle indicates the correlation of the component in PC1 and PC5 at 0.5. (**D**) Decision surface and score plot between principal component 3 (PC3) and component 4 (PC4) to predict the α-glucosidase inhibitory activity. Blue and red surfaces or dots indicate the inactive and active α-glucosidase inhibitory activity. (**E**) Receiving operating characteristic (ROC) of 5-fold cross-validation on the prediction of α-glucosidase inhibitory activity. (**F**) Loading plot of principal component analysis between PC3 to PC4. The blue solid circle indicates the correlation of the component in PC3 and PC4 at 1.0, whereas the red dash circle indicates the correlation of the component in PC3 and PC4 at 0.5.

Figure 3 shows the classification analysis between the active (IC_50_ can be calculated) and inactive (IC_50_ cannot be calculated) classes of α-amylase and α-glucosidase inhibitory activities based on the principal component analysis (PCA) of the phytochemical constituents of 31 TTM samples. The classification analysis against the α-amylase enzyme revealed that the principal components 1 and 5 were best described for the classification of α-amylase inhibitory activity, with an accuracy score of 0.97 (Figure 3A). The classification result against the α-amylase enzyme was confirmed, receiving the operating characteristic (ROC) curve of 5-fold cross-validation of the training set, which yielded the area under the curve (AUC) at 0.68 ± 0.24. The result indicates the sensitivity of prediction for both active and inactive classes of the TTM is acceptable for classifying the IC_50_ against the α-amylase enzyme (Figure 3B). The important phytochemical constituents between PC1 and PC5 are shown in Figure 3C. The alkaloid, xanthones, reducing sugar, and saponins were located outside the red circle (Figure 3C), which presents a high loading score (PC1 and PC5 correlation scores were higher than 0.5). The saponins, xanthones, and reducing sugar influenced the inactive TTM in the upper quadrants of the PCs score plot (Figure 3A,C), whereas the alkaloids and flavonoids could influence the active TTM against the α-amylase enzyme, as it was located in the lower quadrants in Figure 3C.

Figure 3D shows the decision boundary and score plot of classification analysis against the α-glucosidase enzyme. We found that the PC3 and PC4 were the best-described classification between active (red color) and inactive (blue color) α-glucosidase inhibitory activity (Figure 3D), with an accuracy score of 1.0. The classification performance of α-glucosidase inhibitory activity was further evaluated by the receiving operating characteristic (ROC) plot of 5-fold cross-validation, which yielded the area under the curve (AUC) at 0.86 ± 0.08 (Figure 3E). This result indicates that the sensitivity of the model to classify the active and inactive classes of α-glucosidase inhibitory activity was higher than the model to classify the active classes of α-amylase inhibitory activity. It evidenced that the α-glucosidase activity was more correlated with the phytochemical constituents compared with the α-amylase inhibitory activity. The loading plot revealed that the xanthones, tannins, flavonoids, and the reducing sugar were highly correlated with the classification result between the PC3 and PC4 (the correlation score was higher than 0.5) (red circle, Figure 3F). The results indicated that the xanthones and saponins were responsible for the inactive inhibitory activity against the α-glucosidase enzymes (upper and lower quadrants), whereas the tannins, flavonoids, and reducing sugar were responsible for the active inhibitory activity against the α-glucosidase enzyme.

## 4. Discussion

Diabetes mellitus (DM) is a primary global public health issue that leads to life-threatening complications [29]. The various complications associated with DM include nephropathy, neuropathy, cardiovascular and renal complications, retinopathy, food-related disorders, and depression [30]. DM is caused either by: (1) deficiency of insulin secretion, and the damage of pancreatic β cells (type I); or (2) insulin resistance related to the non-use of insulin (type II) [4]. Type 1 DM is an autoimmune disorder that affects pancreatic cells and reduces or impairs insulin production [20]. Type 2 DM is a result of the impairment of insulin secretion by pancreatic β cells, and the inability of insulin-sensitive tissues to respond to insulin [4]. The inabilities to produce or use insulin in the cells result in a high glucose level in the bloodstream. A high level of glucose leads to oxidative stress and disease complications [31]. Reducing glucose in the blood circulation is very important to prevent complications in DM patients.

The main conventional drug classes for the treatment of hyperglycemia include: (1) sulfonylureas (improving the release of insulin from the pancreatic β cells); (2) biguanides and thiazolidinediones (TZDs) (lowering hepatic glucose production); (3) peroxisome proliferator-activated receptor-γ (PPARγ) agonists (stimulating the insulin activity); (4) sodium-glucose transport protein 2 (SGLT2) inhibitors (reducing glucose reabsorption); and (5) α-glucosidase inhibitors (interfering with absorption of glucose in the GI tract) [9]. These classes of drugs are administered as monotherapy, or in combination with other hypoglycemic agents. Severe hypoglycemia, weight gain, lower therapeutic efficacy due to the imbalance of glucose concentrations, ineffective dosage regimen, low potency, and side effects due to lack of target specificity, are the major drawbacks associated with the use of conventional medicines [32].

Recently, many studies have focused on the use of the medicinal plants as a therapeutic adjuvant for the treatment of DM [33]. Phytochemical components such as flavonoids are well known to have low side effects over the synthesis compounds, especially in the inhibition of α-glucosidase or anti α-amylase [11]. Moreover, recent studies have found that the antioxidant effects from plants are the key factors for restoring damaging pancreatic β cells or myocardial cells [31,33]. This finding has unfolded based on the fact that the reactive oxygen species (ROS) are the major threatening species that cause damage to pancreatic β cells and microvascular tissues [31].

ROS is the type of compound that contains reactive oxygen. Examples of ROS, such as superoxide anions (^•^O_2_^–^) or hydroxyl radical (^•^OH), can cause oxidative stress to cells [5]. High levels of ROS can occur by: (1) high production of ROS from the cellular oxidative metabolism in mitochondria; (2) a low level of antioxidant compounds or enzymes (i.e., superoxide dismutase (SOD), catalase, or glutathione in the system [5]; or (3) hyperglycemia in bloodstream promoting the release of interleukin-1 (IL-1), excessive glucose autoxidation, and lipid peroxidation [34]. The polyphenol from plants are secondary metabolites characterized by one or more hydroxyl groups binding to one or more aromatic rings, such as tannins, saponins, flavonoids, or phenolic compounds, and are found to have antioxidant effects [35]. The antioxidant mechanism of plant polyphenol work by giving their electron or hydrogen radical to the ROS species from the stable compounds [36], and lowering the glucose level in the blood circulation resulting in a reduction in oxidative stress from the oxidation process [31,33]. The plants contain both mechanisms, are thus of interest for further drug discovery and development as anti-hyperglycemic agents.

The inhibition of α-amylase and α-glucosidase activity is a commonly known pharmacological action for lowering postprandial hyperglycemia. α-Amylase breaks down the polysaccharide into oligosaccharides and disaccharides in the intestinal tract. Then, α-glucosidase catalyzes hydrolysis to produce monosaccharides such as glucose and fructose from disaccharides. This event prevents the absorption of glucose into the blood circulatory system [37,38]. The strategy to decrease carbohydrate digestibility by inhibiting the activity of these two hydrolyzing enzymes to control blood sugar is considered a prophylactic treatment of type 2 DM [10]. Inhibiting these two enzymes reduces blood glucose and prevents oxidative stress in the pancreatic β cells [31]. Plant polyphenols were reported to attribute to these actions [29,30]. Thus, the consumption of food or indigenous plants rich in α-amylase and α-glucosidase inhibitors are beneficial for the therapy of type II diabetes.

Complementary and alternative medicines have been widely used for the treatment of diabetes mellitus worldwide [39], as well as in Thailand. Traditional Thai medicines (TTM) have long been used for lowering blood sugar [13,40]. In this study, we screened the potential inhibitory activities of selected TTM against α-amylase and α-glucosidase enzymes. Since most enzymes have a characteristic optimum pH at which the velocity of the catalyzed reaction is maximal, so do α-amylase and α-glucosidase. If the pH changes, the ionization of groups, both at the enzyme’s active site and on the substrate, will also change and later affect the rate of binding of the substrate to the active site. The optimal pH of these two enzymes in the in vitro condition is pH 6.9 [41,42,43], which was used in this study.

Thirty-one TTMs have long been used traditionally as folklore medicines in the Northeastern region of Thailand. The use of different plant parts was performed by following traditional Thai medicine based on local wisdom, with the aim of achieving biologically active compounds. *A. marginata* is used for laxative. *A. marginata*, *C. filiformis*, and *H. perforate* are individually used as anti-pyretic. *C. filiformis* is used for diuretic, antihypertension and anti-inflammation. *E. cherrevensis* is used for blood and kidney tonic. *G. cowa* is used for internal bleeding. *G. cowa*, *H. perforate*, and *F. indica* are separately used for anti-diarrhea, food poisoning, and dysentery. *P. debilis* is used for abdominal pain treatment. *P. evecta* is used for galactagogue. *F. indica* is used for anthelmintics. *P. anamense* and *F. indica* are separately used for antipruritic and anti-asthma treatments [44]. Moreover, these plants as well as *G. daltonii*, *P. kesiya*, *C. filiformis*, *C. formosum* spp. *pruniflorum* are reported to possess different degrees of cytotoxicity in the hepatocellular carcinoma HepG2 cells [44,45,46,47,48,49,50,51].

According to the information obtained from the local folk healer, four TTM plants have been used for the treatment of diabetes mellitus, which are *P. anamense*, *L. coromandelica*, *A. marginata*, and *C. tomentosa*. Four other plants are used for anti-dyspepsia and anti-flatulence, which are *G. cowa*, *P. debilis*, *E. cuneatum*, and *C. oblongifolius*. Twenty-one TTM plants are used in traditional medicine and local food recipes, but there are no confirmed reports on the treatment of diabetes mellitus. These latter group of plants are *P. evecta*, *E. cherrevensis*, *F. indica*, *H. perforata*, *R. dumetorum*, *C. filiformis*, *P. kesiya*, *T. loureiri*, *R. succedanea*, *R. javanica*, *C. formosum*, *B. anceps*, *D. castanea*, *E. succirubrum*, *D. winitii*, *B. ovata*, *A. harmandii*, *T. triptera*, *T. mucronata*, *A. pulchellus*, and *G. daltonii*.

The present study used 50% water–ethanol as the solvent for extraction based on the principle of the local wisdom to prepare traditional medicine, with the purpose of achieving biologically active compounds. Traditional medicine can be prepared by boiling herbs in water, or using ethanol to prepare a herbal liqueur, tincture, or elixir [52]. Ethanol has been used as a co-solvent when preparing herbal medicines based on the European monograph of traditional herbal medicine [52] and Thai Herbal Pharmacopeia [53]. If water alone was used in the plant extraction, it would be hard to remove the water without using a high boiling temperature, and potentially degrading some compounds. Ethanol also facilitates the water extraction of phytochemicals from the plant material, resulting in large extraction quantities [54]. Moreover, ethanol is safely edible, leaving a safe-to-use extract for further in vivo or clinical study.

In this study, the ethanolic extract of *P. kesiya* twig possessed the highest potency (lowest IC_50_) against the α-amylase enzyme. The phytochemical constituents found in *P. kesiya* were flavonoids (medium), tannins (medium), and saponins (lowest). *P. kesiya* was previously reported to contain phenolics compounds, which were gallic acid 19.7 mg/g, chlorogenic acid 10.1 mg/g, caffeic acid 5 mg/g, vanillic acid 5 mg/g, coumaric acid 3.1 mg/g crude extract [45]. The multivariate analysis found that flavonoids were responsible for the active α-amylase inhibitory activity. This was confirmed with a previous study that flavonoids from plant extracts could inhibit the α-amylase enzyme [55]. In addition, the tannins in the *Pinus* species were responsible for the activity against α-glucosidase inhibitory activity [56], which can be observed by the inhibitory activity against α-glucosidase of *P. kesiya* (Table 1 and Figure 3D–F). Therefore, it suggests that the ethanolic twig extract of *P. kesiya* could inhibit both α-amylase and α-glucosidase enzymes.

Furthermore, the twig extracts of *P. anamense* and the stem extract of *C. oblongifolius* exerted the highest potency against the α-glucosidase enzyme. The phytochemical profile of *P. anamense* and *C. oblongifolius* extracts were similar, sharing the characteristics of medium alkaloid, medium flavonoids, medium reducing sugar, low-to-medium saponin, and low tannins. Based on the multivariate analysis and network-based correlation analysis, the flavonoids, tannins, and reducing sugar were responsible for the α-glucosidase enzyme, which were confirmed by previous reports on the α-glucosidase inhibitory activities of flavonoids [57], and tannins [56,58,59].

Previous research found that the 3-OH in the C-ring and the catechol group (3′-OH and 4′-OH) in the B-ring of flavonoids contributed to the α-glucosidase inhibitory activity by hydrogen bonding (H-bond) with the active site of α-glucosidase enzymes [57]. Tannins are a group of polyphenolic compounds. Tannic acids are plant polyphenols (plant tannins) found vastly in plants [20]. Tannic acids inhibit α-glucosidase by forming H-bonding and hydrophobic interaction in the active site of α-glucosidase, based on the abundance of phenol rings in its structure [20]. Reducing sugars are monosaccharides, or some disaccharides with a terminal aldehyde group (−HC=O), which can be oxidized to a carboxylate (−COO−) functional group [60]. Despite no studies being reported on the α-glucosidase inhibitory activity of reducing sugar, we found that the presence of this structural group was well correlated to the active inhibitory activity against the α-glucosidase enzyme. This finding, however, needs further proof and more detail.

The -OH substituted at the xanthone core structure was attributed to the inhibitory activity of xanthones against the α-glucosidase activity [17]. The less amount of xanthones was found in the extract samples (Figure 1). Hence, this could be the reason for an inactive inhibitory activity against α-glucosidase enzymes. Saponins are triterpene glycosides-based structures, which are widely distributed in the plant as an active foamy agent. The glycone structures of saponins, especially 28-O-monosides and 3,28-O-bidesmosides, were reported to be contributed to the inhibition of α-glucosidase activity [18]. However, our study showed that a high amount of saponins were less correlated to the inhibition of the α-glucosidase enzyme. It should be noted that many more types of phytochemicals may exist in the extract than those we could identify. Moreover, these extract compositions could synergistically exert positive or negative effects on the inhibition of these two enzymes. The elucidation of the phytochemical constituted in the extract is required in order to determine the structure-activity relationship.

Five TTMs are active against both α-amylase and α-glucosidase enzymes, including the ethanolic extracts from *G. cowa* leaf, *P. debilis* leaf, *P. evecta* leaf, *C. formosum* stem, and the *P. kesiya* twig. These five TTMs share a similar phytochemical profile with medium to high flavonoids, except *G. cowa,* which did not comprise flavonoids in the ethanolic leaf extract. *P. evecta* leaf was previously reported to comprise catechin, caffeic acid, ferulic acid [46], and tannin [46]. Moreover, a previous report revealed that the flavonoids found in the ethyl acetate leaf extract of *G. cowa* exerted a glucosidase inhibitory activity [61]. Thus, the flavonoids in this plant were attributed to both α-amylase and α-glucosidase inhibitory activity. Interestingly, the extracts of *G. cowa*, *P. evecta*, and *P. debilis* were reported to be used for the treatment of diabetes in traditional Thai medicine [61,62], and in Sri Lanka [63]. The xanthones of *Cratoxylum* species exhibited α-glucosidase inhibitory activity [64].

Plants synthesize a wide range of chemical compounds, classified into primary and secondary metabolites. Primary metabolites are essential to plants for proper growth and development. Examples of primary metabolites are chlorophyll, lactic acid, certain amino acids, nucleotides, sugars, carbohydrates, and lipids, which have a primary role in metabolic processes such as photosynthesis, respiration, and nutrient assimilation. Secondary metabolites have an important ecological function. They are naturally present in specific plant taxonomy, stages of plant development, and periods of stress. They protect plants from harmful factors that make them competitive in their environments, including protection against predators, pathogens, or environmental stress (i.e., ultraviolet (UV) irradiation, drought, and extreme temperatures). Some secondary metabolites are related to plant fertilization (attraction of insects for pollination). These conditions lead to biodiversity in the compounds present in the plants [65,66]. Examples of secondary metabolites are glycosides, terpenoids, flavonoids, phenolic and polyphenolic compounds, nitrogen-containing alkaloids, and sulfur-containing compounds. Qualitative phytochemical screening is one of the standard techniques to identify new sources of therapeutic agents present in the plant extracts, and was thus performed in this study. The qualitative phytochemical screening will help to understand a variety of chemical compounds that exist in plants, and help the experimental design for the extraction, purification, and identification of the bioactive compounds. Characterization and evaluation of plants’ phytoconstituents can help explore the evidence to support therapeutic claims of those plants against a myriad of diseases.

Based on the major findings of this study, it can be translated that the use of an ethanol–water extract of *G. cowa* leaf, *P. debilis* leaf, *P. evecta* leaf, *C. formosum* stem, and *P. kesiya* twig could convert into future medicinal meaningful for improving diabetes mellitus, since α-glucosidase and α-amylase are potential targets for lowering postprandial glycemia [67]. The α-glucosidase and α-amylase release in the saliva after the first bite of the meal; therefore, these TTM extracts should be taken between meals, or at the first bite of a meal, similar to acarbose or voglibose dosage regimens [68]. The different parts of the plants used in this study mimicked the local uses of traditional medicine, and we found that the different plant parts (*D. castanea* leaf *versus D. castanea* twig and *P. evecta* leaf *versus P. evecta* rhizome) also differently exhibited their inhibitory activity against the α-amylase enzyme (Table 2). This might be because different plant parts contained different phytochemical components (Table 1). The flavonoids, saponins, and xanthones in *D. castanea* leaf were higher than *D. castanea* twig. The alkaloids, flavonoids, reducing sugars, and xanthones in *P. evecta* rhizome were higher than *P. evecta* leaf, whereas the saponins and tannins in *P. evecta* rhizome were lower than *P. evecta* leaf. Therefore, to achieve these therapeutic effects, the correct plant part containing the active metabolite is also required.

Lastly, the transformation from in vitro to the clinic is also challenging [15]. Most plant-based medicines (i.e., TTMs) are not systematically investigated using in vitro, in vivo, and clinical trials in humans [13,14]. Thus, molecular pharmacological-based studies are essential for providing scientific evidence for these herbal medicines. Moreover, toxicological, and phytochemical screenings are required before clinical trials. Furthermore, proper active metabolite identifications are necessary to provide evidence for the further prediction of therapeutic use. Detailed studies are necessary in relation to: (1) the pharmacokinetic properties of TTMs; (2) formulations of effective dosage forms; and (3) TTMs safety [69]. In addition, possible interactions with other anti-diabetic drugs or foods need to be studied in detail. Those studies may facilitate clinical trials determining the potential of TTMs in diabetes management. Thus, it is of high interest that further research on TTMs could lead to novel therapeutics for diabetes mellitus.

## 5. Conclusions

Our study highlights the potential plants for inhibiting α-amylase and α-glucosidase, hence slowing carbohydrate digestion and reducing postprandial glycemia. The ethanolic extract of *P. keyisa* yielded the lowest IC_50_ against α-amylase, whereas *C. oblongifolius* yielded the lowest IC_50_ against α-glucosidase. In addition, the ethanolic extracts of *P. evecta* leaf, *P. kesiya*, *G. cowa,* *C. formosum*, and *P. debilis* exhibited low IC_50_ values in both α-amylase and α-glucosidase enzymes. The use of correlation-based network analysis and a reinforcement learning model to define the attributed compounds to their activities have been central to this study. The reinforcement learning model revealed that the amounts of alkaloids and flavonoids contributed to the α-amylase inhibitory activity, whereas the amounts of flavonoids, tannins, and reducing sugars contributed to the α-glucosidase inhibitory activity. Taken together, our findings support the medicinal values of *P. kesiya*, *C. oblongifolius*, *P. anamense*, *P. evecta* (leaf), *G. cowa*, *C. formosum* spp. *pruniflorum*, and *P. debilis*. These plants are promising for the next study steps. Further appropriate chemical structure elucidation of the active phytochemical constituents and in vivo experiments are required to assess and evaluate the antidiabetic potential of these plant extracts. The studied plants are promising sources of natural antidiabetic agents in food and pharmaceuticals against key enzymes inhibition.

## Figures and Tables

**Table 1:** Scientific names of Thai indigenous plants and parts used in this study.

Sample No.	Scientific Names	Family	Part Used
1	*Abrus pulchellus* ssp. *mollis*	Fabaceae	Stem
2	*Aganosma marginata* (Roxb.) G. Don	Apocynaceae	Leaf
3	*Artabotrys harmandii* Finet & Gagnep.	Annonaceae	Vine
4	*Bombax anceps*	Bombaceae	Stem
5	*Bridelia ovata* Decne.	Euphorbiaceae	Stem
6	*Cassytha filiformis* L.	Lauraceae	Vine
7	*Catunaregam tomentosa*	Rubiaceae	Stem
8	*Cratoxylum formosum* spp. *pruniflorum*	Hypericaceae	Stem
9	*Croton oblongifolius*	Euphorbiaceae	Stem
10 and 11	*Diospyros castanea* Fletcher	Ebenaceae	Twig and Leaf
12	*Diospyros winitii*	Ebenaceae	Stem
13	*Ellipeiopsis cherrevensis* (Pierre ex Finet & Gagnep.) R.E.Fr.	Annonaceae	Twig
14	*Erythrophleum succirubrum*	Fabaceae	Stem
15	*Erythroxylum cuneatum* (Miq.) Kurz	Erythroxylaceae	Twig
16	*Flacourtia indica* (Burm.f.) Merr.	Flacourtiaceae	Twig
17	*Garcinia cowa* Roxb. ex DC.	Guttiferae	Leaf
18	*Glochidion daltonii* (MÜll. Arg.) Kurz	Euphorbiaceae	Stem
19	*Harrisonia perforata* (Blanco) Merr.	Simaroubaceae	Twig
20	*Lannea coromandelica*	Anacardiaceae	Stem
21	*Parinari anamense* Hance	Chrysobalanaceae	Twig
22	*Pinus kesiya* Royle	Pinaceae	Twig
23	*Polyalthia debilis* (Pierre) Finet & Gagnep.	Annonaceae	Leaf
24 and 25	*Polyalthia evecta* (Pierre) Finet & Gagnep.	Annonaceae	Rhizome and Leaf
26	*Rhodamnia dumetorum* (DC.) Merr. & L.M. Perry	Myrtaceae	Twig
27	*Rhus javanica*	Anacardiaceae	Stem
28	*Rhus succedanea*	Anacardiaceae	Twig
29	*Terminalia mucronata*	Combretaceae	Stem
30	*Terminalia triptera*	Combretaceae	Stem
31	*Tetracera loureiri* (Finet. & Gagnep.) Pierre ex Craib	Dilleniaceae	Stem

**Table 2:** α-Amylase and α-glucosidase inhibitory activities of 31 TTM plant samples. The E_max_ is the maximum %inhibitory activity of the highest concentration of TTM extract. The maximum concentrations used for the inhibition of α-amylase and α-glucosidase were 2.75 µg/mL and 0.67 µg/mL, respectively. IC_50_ is the 50% inhibition concentration of TTM.

Scientific Names	α-Amylase		α-Glucosidase	
	E_max_	IC_50_ (µg/mL)	E_max_	IC_50_ (µg/mL)
*Abrus pulchellus* ssp. *mollis*	24.7 ± 0.3 ^e^	ND	15.9 ± 0.1 ^o^	ND
*Aganosma marginata*	16 ± 0.1 ^c^	ND	37.6 ± 0.2 ^h,i^	ND
*Artabotrys harmandii*	27.2 ± 0.2 ^f^	ND	30.8 ± 0.2 ^l^	ND
*Bombax anceps*	13.2 ± 0.2 ^b^	ND	39.1 ± 0.6 ^e,f,g^	ND
*Bridelia ovata*	92.3 ± 0.4 ^q^	0.16 ± 0.004 ^g,h^	32.7 ± 1.4 ^j,k^	ND
*Cassytha filiformis*	35.6 ± 0.3 ^h,i^	c	36.7 ± 0.4 ^i^	ND
*Catunaregam tomentosa*	21.3 ± 2.2 ^p^	ND	33.9 ± 0.4 ^j^	ND
*Cratoxylum formosum* spp. *pruniflorum*	87 ± 0.2 ^f^	0.17 ± 0.005 ^g,h,i^	50.3 ± 0.4 ^c,d^	0.65 ± 0.03 ^b,c^
*Croton oblongifolius*	12.6 ± 0.4 ^a^	ND	53.0 ± 0.7 ^a,b^	0.49 ± 0.05 ^a^
*Diospyros castanea* (leaf)	84.1 ± 0.2 ^m^	0.16 ± 0.0007 ^g,h^	20.9 ± 0.2 ^n^	ND
*Diospyros castanea* (twig)	42.1 ± 0.4 ^i^	ND	29.7 ± 0.2 ^m^	ND
*Diospyros winitii*	93.3 ± 0.1 ^r^	0.12 ± 0.0004 ^b^	39.7 ± 0.2 ^e,f^	ND
*Ellipeiopsis cherrevensis*	77.8 ± 0.2 ^k^	0.18 ± 0.005 ^a^	39.8 ± 0.3 ^e,f^	ND
*Erythrophleum succirubrum*	35.4 ± 0.2 ^h,i^	ND	32.5 ± 0.2 ^j,k^	ND
*Erythroxylum cuneatum*	86.5 ± 0.1 ^o^	0.17 ± 0.011 ^h,i^	39.6 ± 0.1 ^e,f^	ND
*Flacourtia indica*	84.4 ± 0.1 ^n^	0.16 ± 0.004 ^f,g,h^	39.1 ± 0.2 ^f,g^	ND
*Garcinia cowa*	99.6 ± 0.2 ^u^	0.13 ± 0.001 ^c^	51.1 ± 0.4 ^c^	0.63 ± 0.02 ^b,c^
*Glochidion daltonii*	27.3 ± 2.1 ^p,q,r^	ND	37.6 ± 0.2 ^g,h,i^	ND
*Harrisonia perforata*	35.2 ± 0.3 ^h,i^	ND	39.9 ± 0.7 ^d,e,f^	ND
*Lannea coromandelica*	29.9 ± 0.2 ^f,g^	ND	31.4 ± 0.2 ^k^	ND
*Parinari anamense*	35.1 ± 0.3 ^h,i^	ND	54.5 ± 0.5 ^a^	0.52 ± 0.01 ^a^
*Pinus kesiya*	94.9 ± 0.1 ^t^	0.11 ± 0.001 ^a^	52.3 ± 0 ^a,b^	0.60 ± 0.01 ^b,c^
*Polyalthia debilis*	99.5 ± 0.2 ^u^	0.14 ± 0.0001 ^d,e,f^	50.0 ± 0.1 ^c,d,e^	0.72 ± 0.02 ^c^
*Polyalthia evecta* (leaf)	93.9 ± 0 ^s^	0.14 ± 0.0013 ^c,d^	52.0 ± 0.2 ^b^	0.57 ± 0.00 ^a,b^
*Polyalthia evecta* (rhizome)	35.8 ± 0.6 ^h,i^	ND	50.6 ± 0.3 ^c^	0.63 ± 0.03 ^b,c^
*Rhodamnia dumetorum*	30.3 ± 0.2 ^g^	ND	10.5 ± 0.8 ^p^	ND
*Rhus javanica*	82.1 ± 0.0 ^l^	0.14 ± 0.0009 ^c,d,e^	38.9 ± 0.1 ^f,g,h^	ND
*Rhus succedanea*	29.9 ± 0.1 ^f,g^	ND	31.6 ± 0.2 ^j,k^	ND
*Tetracera loureiri*	47.3 ± 0.1 ^j^	ND	20.5 ± 0.2 ^n^	ND
*Terminalia mucronata*	95.7 ± 0.1 ^u^	0.16 ± 0.001 ^e,f,g^	9.7 ± 0.5 ^p^	ND
*Terminalia triptera*	33.4 ± 0.1 ^h^	ND	32.6 ± 0.2 ^j,k^	ND

ND: cannot be determined because the %inhibition was less than 50%. **IC_50_**: the half-maximal inhibitory concentration of TTM extracts. **E_max_**: the maximum inhibitory activity (percentage of inhibition) of the highest concentration of TTM extracts. Different lower-case letters indicate a significant difference of data among different plant samples in the same column at *p* < 0.05.

## Data Availability

All data and source codes used in this manuscript were available on: https://github.com/TarapongSrisongkram/antihyperglycemia_Thaiherbs (accessed on 6 January 2022).

## Supplementary Materials

The following supporting information can be downloaded at: https://www.mdpi.com/article/10.3390/nu14020267/s1, Table S1: Spearman’s correlation results with a *p*-value of the percentage inhibitions between phytochemical components and α-amylase and α-glucosidase inhibitory activities.
