# Supplementary material for: Machine Learning and In Vitro Chemical Screening of Potential α-Amylase and α-Glucosidase Inhibitors from Thai Indigenous Plants
Source: Nutrients. 2022 Jan 9;14(2):267. doi: 10.3390/nu14020267 (PMC8781461; doi:10.3390/nu14020267)
Supplement: Supplementary file 1 [file nutrients-14-00267-s001.zip › nutrients-1507398-supplementary.pdf]

Supplementary data

Machine Learning and *in Vitro* Chemical Screening of Potential α-Amylase and α-Glucosidase Inhibitors from Thai Indigenous Plants

Tarapong Srisongkram[1,2], Sasisom Waithong[3], Thaweesak Thitimetharoch[4] and Natthida Weerapreeyakul[1,2*]

1 Division of Pharmaceutical Chemistry, Faculty of Pharmaceutical Sciences, Khon Kaen University, Khon Kaen 40002 and Thailand; tarasri@kku.ac.th (T.S.); natthida@kku.ac.th (N.W.)
2 Human High Performance and Health Promotion Research Institute, Khon Kaen University, Khon Kaen 40002, Thailand; tarasri@kku.ac.th (T.S.); natthida@kku.ac.th (N.W.)
3 Graduate School (in the Program of Aesthetic Sciences and Health), Faculty of Pharmaceutical Sciences, Khon Kaen University, Khon Kaen 40002, Thailand; namwansasi@gmail.com (S.W.)
4 Division of Pharmacognosy and Toxicology, Faculty of Pharmaceutical Sciences, Khon Kaen University, Khon Kaen 40002, Thailand; thathi@kku.ac.th
* Correspondence: natthida@kku.ac.th (N.W.); Tel.: 66-43-202-378

Supplementary Table S1: Spearman's correlation results with a p-value of the percentage inhibitions between phytochemical components and α-amylase and α-glucosidase inhibitory activities.

	Emax_amylase	Emax_glucosidase	IC_{50}_amylase	IC_{50}_glucosidase	Alkaloids	Antaquinones	Carotenoids	flavonoids	Reducing sugars	Saponins	Tannins	Xanthones
**Emax_amylase**	1.0***	0.251	-0.589*	0.635	-0.1	0.122	0.061	0.113	-0.268	-0.34	-0.122	0.158
**Emax_glucosidase**		1.0***	-0.396	-0.946***	0.274	0.051	0.122	0.384*	0.338	-0.406*	0.407*	-0.012
**IC_{50}_amylase**			1.0***	0.41	0.498	0.118	0.473	-0.282	-0.029	0.099	-0.079	-0.091
**IC_{50}_glucosidase**				1.0***	-0.165	NaN	NaN	0.353	-0.165	-0.736*	-0.477	0.251
**Alkaloids**					1.0***	0.205	0.338	-0.051	0.254	-0.139	0.104	-0.025
**Antaquinones**						1.0***	-0.033	0.127	0.31	-0.104	0.158	-0.089
**Carotenoids**							1.0***	-0.085	-0.108	0.042	-0.169	-0.089
**flavonoids**								1.0***	0.548**	-0.369*	-0.043	-0.004
**Reducing sugars**									1.0***	-0.311	0.191	-0.084
**Saponins**										1.0***	-0.167	-0.196
**Tannins**											1.0***	-0.311
**Xanthones**												1.0***

*, **, *** are p-value less than 0.05, 0.01, 0.001, respectively.
NaN means correlation cannot be calculated.
